# Postmarket Safety Surveillance of FDA-Cleared Prescription Digital Therapeutics Using the Manufacturer and User Facility Device Experience (MAUDE) Database: A Pharmacovigilance Study

**DOI:** 10.7759/cureus.85343

**Published:** 2025-06-04

**Authors:** Shaheen E Lakhan

**Affiliations:** 1 Medical Office, Click Therapeutics, Inc., New York City, USA; 2 Neurology, Western University of Health Sciences, Pomona, USA; 3 Neurology, A.T. Still University School of Osteopathic Medicine in Arizona, Mesa, USA; 4 Neurology, Morehouse School of Medicine, Atlanta, USA; 5 Bioscience, Global Neuroscience Initiative Foundation, Miami, USA

**Keywords:** adverse event reporting, digital health, endeavorrx, fda, maude database, pdt, postmarket surveillance, prescription digital therapeutics, software as a medical device, somryst

## Abstract

Background and aim

Prescription digital therapeutics (PDTs) are software-based medical treatments authorized by the U.S. Food and Drug Administration (FDA) for a range of chronic conditions. As clinical adoption increases, postmarket surveillance becomes essential to ensure safety beyond controlled trial environments. This study aimed to characterize postmarket adverse events and product problems associated with FDA-cleared PDTs using data from the FDA's Manufacturer and User Facility Device Experience (MAUDE) database. This approach supports the generation of hypothesis-forming safety signals in naturalistic care settings, where adherence patterns, off-label use, and user variability are more likely to emerge.

Methods

We conducted a retrospective, pharmacovigilance study of the MAUDE database through April 30, 2025. Device-specific product codes were used to identify reports related to 13 FDA-cleared PDTs as follows: reSET (Middletown, CT: PursueCare) for substance use disorder, reSET-O (Middletown, CT: PursueCare) for opioid use disorder, Somryst (Alpharetta, GA: Nox Health) and SleepioRx (San Francisco, CA: Big Health) for chronic insomnia, EndeavorRx (Boston, MA: Akili, Inc.) for pediatric attention-deficit/hyperactivity disorder (ADHD), Parallel (San Francisco, CA: Mahana Therapeutics Inc.) and Regulora (Chicago, IL: metaMe Health) for irritable bowel syndrome, Stanza (San Francisco, CA: Swing Therapeutics, Inc.) for fibromyalgia, AspyreRx (New York City, NY: Click Therapeutics, Inc.) for type-2 diabetes, Rejoyn (Princeton, NJ: Otsuka Precision Health, Inc.) for major depressive disorder, DaylightRx (San Francisco, CA: Big Health) for generalized anxiety disorder, MamaLift Plus (Princeton, NJ: Curio Digital Therapeutics) for postpartum depression, and CT-132 (New York City, NY: Click Therapeutics, Inc.) for episodic migraine. Each report was manually reviewed for relevance and data extracted on event type, patient demographics, outcomes, and product details.

Results

Four reports were initially identified. Two were excluded after verification that they involved non-cleared or over-the-counter digital products. Of the remaining two, one report involved Somryst for insomnia prescribed to a patient with a seizure disorder, a known contraindication listed in its labeling, and resulted in disability. The other involved EndeavorRx, which reported perceived ineffectiveness without clinical harm. No adverse events were identified for the remaining 11 PDTs.

Conclusions

In this study, only two adverse event reports associated with FDA-cleared PDTs were identified in the MAUDE database. While this may suggest a favorable safety profile, the findings must be interpreted with caution given the limitations of passive surveillance systems, including underreporting, reporting bias, and limited capacity to capture software-specific harms. These preliminary results highlight the need for modernized, active safety monitoring frameworks tailored to the unique risks of digital therapeutics.

## Introduction

Prescription digital therapeutics (PDTs) are a new class of medical devices that deliver evidence-based interventions via software. Cleared by the U.S. Food and Drug Administration (FDA) through either the 510(k) or De Novo pathways, PDTs have gained traction for managing conditions such as major depressive disorder, substance use disorder, chronic insomnia, attention-deficit/hyperactivity disorder (ADHD), and irritable bowel syndrome [1]. While pivotal trials provide initial efficacy and safety data, postmarket surveillance remains a vital component of their regulatory lifecycle.

The FDA’s Manufacturer and User Facility Device Experience (MAUDE) database allows for voluntary and mandatory reporting of adverse events, malfunctions, and device-related problems [2]. A recent analysis of the MAUDE database from September 2019 to December 2022 found over 4.4 million initial reports submitted by manufacturers, with device malfunctions accounting for approximately two-thirds of the total [3]. Injuries were reported in more than 1.5 million cases, while deaths were identified in just over 13,500 reports. The study also found that although most reports (71%) were submitted within the FDA’s 30-day deadline, a significant proportion were delayed or lacked valid date information. These findings underscore both the scale and challenges of medical device surveillance using MAUDE, including issues with timeliness and data completeness.

This study aimed to identify and characterize adverse events and product problems associated with FDA-cleared PDTs as reported in the MAUDE database. The objective was to evaluate the postmarket safety profile of these software-based medical treatments and to assess the adequacy of existing surveillance tools in capturing digital-specific risks.

## Materials and methods

We conducted a regulatory database-based, retrospective, pharmacovigilance study using the FDA’s MAUDE database [2]. The search was conducted through April 30, 2025. To identify relevant adverse event reports, we queried the database using the exhaustive list of FDA-assigned product codes as follows: PWE, QVO, QFT, QMY, QWI, QXC, SAP, SCP, and SEE. These codes correspond to the 13 FDA-cleared PDTs included in this analysis (in order of first to last clearance) as follows: reSET for substance use disorder (Middletown, CT: PursueCare) [4], reSET-O for opioid use disorder (Middletown, CT: PursueCare) [5], Somryst for chronic insomnia (Alpharetta, GA: Nox Health) [6], EndeavorRx for pediatric ADHD (Boston, MA: Akili, Inc.) [7], Parallel for irritable bowel syndrome (San Francisco, CA: Mahana Therapeutics Inc.) [8], Regulora for irritable bowel syndrome (Chicago, IL: metaMe Health) [9], Stanza for fibromyalgia (San Francisco, CA: Swing Therapeutics, Inc.) [10], AspyreRx for type-2 diabetes (New York City, NY: Click Therapeutics, Inc.) [11], Rejoyn for major depressive disorder (Princeton, NJ: Otsuka Precision Health, Inc.) [12], DaylightRx for generalized anxiety disorder (San Francisco, CA: Big Health) [13], MamaLift Plus for postpartum depression (Princeton, NJ: Curio Digital Therapeutics) [14], SleepioRx for chronic insomnia (San Francisco, CA: Big Health) [15], and CT-132 for episodic migraine (New York City, NY: Click Therapeutics, Inc.) [16].

Reports were included if they contained a matching product code and manual review confirmed the device as a cleared, prescription-only PDT authorized by the FDA. Reports were excluded if they referred to over-the-counter, investigational, or non-prescription software products, if they lacked sufficient detail to confirm regulatory clearance, or if they were misclassified under a shared product code used by multiple device types. Each included report was reviewed manually, and data were extracted on device name, product code, event type, patient demographics (when available), outcome, and narrative description. As this study involved secondary analysis of publicly available, de-identified data from a federal regulatory database, it was exempt from institutional review board (IRB) approval.

## Results

A total of four MAUDE reports matched the search criteria based on product codes. Upon manual review, two of these were found to involve non-cleared products - the Autonomous User Rehabilitation Agent and EndeavorOTC - and were excluded from further analysis. The remaining two reports, both involving FDA-cleared PDTs, were included in the final dataset (Table 1).

**Table 1:** Summary of MAUDE reports involving FDA-cleared PDTs. Summary of adverse event reports from the FDA's Manufacturer and User Facility Device Experience (MAUDE) database involving cleared prescription digital therapeutics (PDTs) through April 2025. QVO and QFT are FDA product codes representing specific device categories. Event type indicates the classification of the report in MAUDE. Patient description includes relevant demographic or clinical context. Outcome denotes reported health impact. The report date is when the event was entered into the MAUDE database. The clearance date is the official FDA marketing authorization date for the PDT. Somryst (Alpharetta, GA: Nox Health); EndeavorRx (Boston, MA: Akili, Inc.)

PDT name	Product code	Event type	Patient description	Outcome	Report date	Clearance date
Somryst	QVO	Injury	35F with seizure disorder	Disability	May 22, 2022	March 23, 2020
EndeavorRx	QFT	Malfunction	Male user, ineffective use	No harm	October 26, 2023	June 15, 2020

Out of the total search results, only two MAUDE reports were verified as involving cleared PDTs.

PDT name: Somryst, product code: QVO

An injury report involving a 35-year-old female with a known seizure disorder. The patient reported muscular rigidity, anxiety, neck pain, sleep dysfunction, and seizures after use. The reporter claimed the device was prescribed despite contraindications. The outcome was listed as disability. Note, Somryst received FDA clearance on March 23, 2020.

PDT name: EndeavorRx, product code: QFT

A malfunction report submitted by a male user indicates a lack of therapeutic effect. The report noted no clinical symptoms or physical harm. Note, EndeavorRx was cleared by the FDA on June 15, 2020. No deaths, hospitalizations, or life-threatening adverse outcomes were reported for any cleared PDT in the dataset. Further, no adverse events were reported in MAUDE for the other 11 cleared PDTs as follows: reSET, reSET-O, DaylightRx, Parallel, Regulora, AspyreRx, MamaLift Plus, and CT-132. The earliest PDT in the dataset, reSET, was cleared on September 14, 2017, and the latest, CT-132, on April 11, 2025.

## Discussion

This study of the FDA’s MAUDE database identified only two adverse event reports linked to the 13 FDA-cleared PDTs evaluated [4-16]. The first involved Somryst, a PDT incorporating cognitive behavioral therapy for insomnia (CBT-I), in a patient with a known history of epilepsy [6]. This condition is explicitly listed as a contraindication in the product’s labeling, indicating that the adverse outcome stemmed from off-label use rather than product malfunction [17]. This report underscores the importance of prescriber adherence to labeling, particularly for software-based therapeutics where contraindications may be less familiar to clinicians. The reported event, which included muscular rigidity, anxiety, and seizures, resulted in disability and suggests that real-world clinical use may sometimes deviate from approved indications, with potentially serious consequences. This finding parallels earlier safety concerns observed with other digital or device-based therapeutics used in contraindicated populations, reinforcing the ongoing need for provider education and label-informed decision-making [1,2].

The second report pertained to EndeavorRx, a PDT for pediatric ADHD, which was associated not with physical harm but with perceived therapeutic ineffectiveness [7]. Although the absence of clinical injury is reassuring, perceived ineffectiveness can erode patient and caregiver confidence and compromise adherence. In digital therapeutics, where efficacy is closely tied to user engagement and behavioral activation, patient-reported outcomes such as disengagement, dissatisfaction, or lack of improvement may serve as early indicators of therapeutic mismatch or usability issues [18]. This highlights the evolving definition of what constitutes an “adverse event” in the context of software-based interventions, which may demand new frameworks for capturing non-traditional harms.

Notably, two additional reports retrieved in the initial query, Autonomous User Rehabilitation Agent (reported under product code PWE) and EndeavorOTC (QFT), were excluded upon verification that these were not for FDA-cleared, prescription products [19,20]. Their preliminary inclusion underscores the need for meticulous review of MAUDE entries when conducting postmarket analyses, particularly in categories like software as a medical device, where overlapping product codes, brand extensions, or investigational tools can be easily conflated with cleared and prescribed devices. This is not merely a technical concern; failure to verify regulatory status could distort risk assessments and undermine confidence in safety analyses.

When viewed in contrast to traditional implantable or hardware-based devices, which collectively account for tens of thousands of MAUDE reports annually, the very low incidence of adverse event reporting among PDTs may suggest a reassuring safety profile [3]. However, such an interpretation should be approached with caution. Digital therapeutics are fundamentally distinct from mechanical devices in that they do not pose the same direct physical risks, but they introduce unique failure modes. These may include software bugs, algorithmic bias, data corruption, ineffective engagement strategies, and behavioral side effects, none of which are well captured by the existing adverse event reporting infrastructure. Indeed, traditional surveillance systems like MAUDE were designed for tangible device malfunctions, not for nuanced failures of engagement, cognition, or symptom control inherent to digital therapies [21].

Moreover, underreporting is a well-known limitation of MAUDE [3]. While manufacturers and user facilities are required to report certain types of events, patients, clinicians, and caregivers are often unaware of their ability to voluntarily submit reports, or may not recognize digital malfunctions as reportable occurrences. The sporadic and subjective nature of digital therapeutic engagement also makes attribution challenging. For example, if a patient discontinues a PDT due to boredom or lack of perceived benefit, this may never be documented, despite potentially reflecting a systemic usability flaw.

It is also plausible that the limited number of reports reflects low utilization rates for many PDTs, especially those recently cleared. The FDA has only begun authorizing PDTs in recent years, and many remain in early commercialization stages or are limited to specific healthcare systems. Thus, the low signal in MAUDE could be a function of market immaturity rather than inherent safety.

Nonetheless, these findings reinforce the imperative to modernize postmarket safety surveillance for digital therapeutics. Real-world evidence networks, app-based analytics, and integration with electronic health records offer promising pathways for supplementing passive reporting systems like MAUDE. Regulatory proposals have called for active postmarket monitoring frameworks tailored to regulated digital therapeutics, including real-time usage metrics, dropout rates, error logs, and engagement biomarkers as proxies for efficacy and safety [22]. Standardizing these approaches and incorporating them into postmarket regulatory expectations would mark a meaningful advance in digital therapeutic oversight.

Equally important is the refinement of device classification systems and product code assignments within the FDA. Current codes often group heterogeneous products, including investigational, consumer-grade, and cleared therapeutics, under similar identifiers, complicating surveillance queries and interpretation [2]. A more granular, indication-specific taxonomy for digital products would improve signal detection and public transparency.

This study offers several methodological and conceptual strengths that contribute meaningfully to the growing body of literature on digital therapeutic safety. First, it represents the most inclusive assessment of postmarket safety signals across the entire landscape of FDA-cleared PDTs to date, spanning diverse therapeutic areas including psychiatry, neurology, gastroenterology, and endocrinology. By systematically evaluating all PDTs cleared through April 2025, the study enables cross-comparison and signal detection within and across indications. Second, the use of the FDA’s MAUDE database as a primary data source allows for real-world safety surveillance beyond the constraints of controlled clinical trials. This approach supports the generation of hypothesis-forming safety signals in naturalistic care settings, where adherence patterns, off-label use, and user variability are more likely to emerge. Third, the study employed rigorous manual verification of each report to confirm device identity, regulatory status, and clinical relevance, an essential step given the frequent misclassification of software products and the overlapping nomenclature in FDA product codes. Finally, by highlighting both the scarcity of adverse event reports and the limitations of current surveillance infrastructure, the study advances an emerging regulatory discourse on the need for purpose-built frameworks to monitor software-based therapeutics in the postmarket phase.

In short, while this analysis supports a preliminary favorable safety profile for FDA-cleared PDTs, it also illustrates the limitations of current surveillance tools in capturing the full spectrum of clinically meaningful risks posed by digital interventions.

Limitations

This study has several limitations inherent to both the MAUDE database and the evolving nature of PDTs. First, the voluntary nature of reporting by clinicians, patients, and caregivers introduces a high likelihood of underreporting, particularly for subjective or non-severe events. Second, many submitted reports are incomplete, unverified, or lack sufficient clinical detail to assess causality. The database does not standardize terminology, nor does it require validation or follow-up by FDA reviewers, limiting its utility for rigorous safety profiling.

Additionally, we did not control for or estimate usage rates of individual PDTs. The exposure denominator is unknown, some products may have had very limited distribution during the study period, thereby lowering the probability of adverse event capture. Misclassification is also possible, given overlapping product codes and brand naming conventions. Although every effort was made to exclude non-cleared devices, the risk of inclusion or exclusion bias remains.

Importantly, the MAUDE database is not designed for pharmacovigilance of behavioral or cognitive interventions and may not adequately reflect the risk profile of software-based therapeutics. Many adverse effects relevant to digital health, such as disengagement, increased anxiety, or digital fatigue, are unlikely to be captured under current reporting frameworks.

Lastly, the exclusion of investigational or consumer-facing products, while necessary to focus on FDA-cleared therapeutics, narrows the scope of generalizability. Future research should explore integrated data streams, including claims data, usage analytics, clinical outcomes, and real-world effectiveness studies, to develop a more complete picture of PDT safety in practice.

## Conclusions

This regulatory database-based retrospective pharmacovigilance study identified only two adverse events associated with FDA-cleared PDTs. While this may suggest a favorable postmarket safety profile, this should be interpreted with caution due to known limitations of passive surveillance systems. These include underreporting, reporting bias, and insufficient mechanisms for capturing software-specific risks. The findings highlight the need for modernized surveillance frameworks tailored to digital therapeutics, incorporating active safety monitoring methods and real-world engagement data to ensure comprehensive postmarket oversight.
